# Hepatocyte growth factor may contribute to male protection against pulmonary arterial hypertension

**DOI:** 10.1186/s13293-026-00852-6

**Published:** 2026-03-01

**Authors:** Lejla Medzikovic, Grégoire Ruffenach, Ateyeh Dehghanitafti, Brenda Wong, Ashley Ryder, Mohammad Reza Hatamnejad, Wasila Sun, Leana Esdin, Joshua Eghbali, Adam Brownstein, Asif Razee, Soban Umar, Jason Hong, Mansoureh Eghbali

**Affiliations:** 1 Division of Molecular Medicine, Department of Anesthesiology and Perioperative Medicine, David Geffen School of Medicine, University of California Los Angeles, BH-550 CHS, Los Angeles, CA 90095-7115 USA; 2 Hypertension Pulmonaire : Physiopathologie and Innovation Thérapeutique (HPPIT), Université Paris-Saclay, Inserm, UMR_S 999, AP-HP, Hôpital Bicêtre, Hôpital Marie Lannelongue (Groupe Hospitalier Paris Saint Joseph), ERN-LUNG, Le Plessis Robinson, France; 3 Division of Pulmonary and Critical Care Medicine, David Geffen School of Medicine, University of California Los Angeles, Los Angeles, CA USA

## Abstract

**Background:**

Pulmonary arterial hypertension (PAH) presents as increased pressure in the pulmonary arteries (PA) leading to cardiac right ventricular (RV) failure and death. Pulmonary arterial (PA) remodeling characterized by enhanced proliferation of pulmonary arterial smooth muscle cells (PASMC) and fibroblasts (PAFB) underlies PAH. There are currently no cures and PAH mortality remains high. PAH has a striking female-predominant incidence – 4:1 ratio – indicating that males may have a protective factor. However, to date only a few sex-biased factors in PAH have been investigated.

**Methods:**

Analyses were performed on a publicly available microarray dataset (GSE117261), comprising human lung tissues from PAH patients and healthy controls, as well as publicly available single-cell lung atlases of humans and mice. Lung tissue, plasma, PASMC and PAFB were collected from male and female PAH patients. Cell proliferation was assessed after recombinant HGF protein stimulation. PH was induced in male and female rats by monocrotaline (MCT). Lung-specific knockdown was performed by intratracheal siRNA instillation the first two weeks after MCT injection. PA and RV function were assessed by echocardiography, RV systolic pressure by catheterization, and PA remodeling by histology.

**Results:**

HGF was only upregulated in lungs of male PAH patients compared to male control lungs, but not in female PAH patients vs. female controls. Elevated plasma HGF correlated with favorable clinical characteristics only in male PAH patients. HGF is highly expressed in vascular SMC and FB in the lung and recombinant HGF inhibited PASMC and PAFB proliferation to a greater extent in cells isolated from male PAH patients compared to female. Lung HGF expression is increased to a higher extent and longer duration at early stage of PH in male rats in MCT model vs. female rats. Finally, knockdown of HGF in the lungs in early disease stage exacerbated PH in male rats characterized by higher mortality, worsened RV and PA function as well as enhanced PA medial thickening and adventitial fibrosis.

**Conclusions:**

Lung HGF expression may be upregulated to counteract PAH disease progression by inhibiting proliferation of PASMC and PAFB. Elevated HGF in males might at least partially account for the lower incidence of male PAH patients.

**Supplementary Information:**

The online version contains supplementary material available at 10.1186/s13293-026-00852-6.

## Plain Language Summary

Pulmonary arterial hypertension (PAH) is a deadly disease where the pressure in lung vessels is chronically elevated. There are up to 4 times more female than male PAH patients, which can mean that males have a protective factor. By analyzing publicly available bioinformatic datasets, we found that the gene HGF, coding for hepatocyte growth factor, is expressed at higher levels in the lungs of male PAH compared to healthy male lungs but did not find this difference in lungs of female PAH patients compared to healthy female lungs. We observed that higher HGF in the blood of male patients correlates with better lung function, but not in females. In the lungs, HGF is expressed in cells that are known to play a key role in PAH development: smooth muscle cells and fibroblasts. We found that HGF protects against dysfunction of these cells but observed that the effect is larger in cells from male PAH patients than female PAH patients. In a rat model of experimental PH, we found that HGF expression in the lung is essential for protection against PAH disease progression. Together our data demonstrates that higher HGF levels in males may be one of the reasons why males are less susceptible to develop PAH than females.

**Supplementary Information:**

The online version contains supplementary material available at 10.1186/s13293-026-00852-6.

## Highlights

HGF is expressed at higher levels only in the lungs of male PAH patients compared to healthy male lungs, but not in females.

Higher plasma HGF levels correlate with favorable clinical characteristics only in male PAH patients.

HGF inhibits proliferation of PASMC and PAFB isolated from male PAH patients to a greater extent than cells from female PAH patients.

HGF is upregulated to a higher and longer extent in lungs of male than female rats upon MCT PH stimulus.

Knockdown of HGF in the lungs early after MCT injection exacerbates PH progression in male rats.

**Supplementary Information:**

The online version contains supplementary material available at 10.1186/s13293-026-00852-6.

## Background

Pulmonary arterial hypertension (PAH) is a devastating pulmonary vascular lung disease characterized by chronically increased pressure in the pulmonary arteries (PA) leading to cardiac right ventricular (RV) failure, and ultimately death [1]. A hallmark pathophysiological mechanism underlying PAH is pulmonary artery vascular remodeling where smooth muscle cells (PASMC) and fibroblasts (PAFB) adopt proliferative phenotypes [2, 3]. While this devastating disease is relatively rare, affecting 15–50 people per million, there is currently no cure, and patients eventually require lung and heart transplantation [1]. Indeed, despite current therapeutic advances, the 3-year-mortality after PAH diagnosis is still as high as 55% depending on the risk category [4]. This dire statistic reflects essential concepts of PAH pathogenesis and pathophysiology, and thus effective therapy, are yet unknown.

Strikingly, the incidence of PAH is much higher in female patients, up to a 4:1 ratio as compared to male PAH patients [5], indicating that males may have a protective factor that could help elucidate the pathogenesis of PAH. Indeed, sex differences in key PAH pathophysiological mechanisms have been reported as PASMC from female PAH patients were shown to possess higher proliferation rates than PASMC isolated from male PAH patients [6]. Additionally, female PAFB were reported to be more activated than male PAFB [7]. However, to date only a few sex-biased factors in PAH have been investigated, with most research efforts directed against the role of sex hormones in PAH [8–11]. 

In the present study, we employed an unbiased bioinformatic and experimental approach to elucidate genes expressed in a sex-dimorphic manner in PAH patient lungs and identified hepatocyte growth factor (HGF). While sex differences in HGF expression have been reported in arthritic diseases and cancer [12–14] and HGF has previously been shown to attenuate PH in rat models [15–18], nothing is known about the role of HGF in sex differences underlying PAH. In this study, we demonstrate that HGF may be upregulated as a protective mechanism against PAH in males.

## Methods

### Bioinformatics

We re-examined publicly available microarray dataset GSE117261 from the Pulmonary Hypertension Breaktrough Initiative (PHBI) consisting of lungs from 58 PAH patients and 25 control lung tissues, together with all available clinical information [19]. Differential expression analysis was performed using the R limma package (R Core Team (2018)) [20]. Genes were considered differentially expressed for an adjusted *P* value < 0.05 and an absolute fold change > 1.5. First, samples were stratified by sex, enabling a comparison between PAH patients and healthy subjects within each sex. Subsequently, using Spearman rank correlation, we correlated the fold-change and statistical significance of DEGs gene between males and females to pinpoint genes exhibiting differential variation in PAH. Subsequently, to elucidate the clinical relevance of sex-specific gene expression patterns, genes displaying sex-specific differential expression in PAH patients were correlated with clinical parameters, including age, mean pulmonary arterial pressure (mPAP), cardiac output (CO), cardiac index, pulmonary vascular resistance (PVR), B-type natriuretic peptide (BNP) levels, and six-minute walk distance (6MWD). Lastly, a two-way ANOVA analysis was employed to identify genes that were: (1) not significantly differentially expressed in an overall analysis of control vs. PAH lungs, and (2) significantly differentially expressed between control and PAH only in one of the sexes.

### Human lung samples

Explanted lung tissue samples were collected from patients at UCLA and the PHBI. Informed consent was given with approval by the local ethics committees. Normal lung tissue was obtained from control patients who either had a diagnosis of pneumothorax or adenocarcinomas. Patient characteristics are described in Table 1.

**Table 1:** Characteristics of the PHBI and UCLA lung tissue cohorts stratified by sex. Available data is presented as median + interquartile range for continuous data, or as count + percentage for categorical data. Continuous data tested with Mann-Whitney test. Categorical data tested with fisher’s exact test. 6MWD: 6-minute walk distance, CO: cardiac output, mPAP: mean pulmonary arterial pressure, PVR: pulmonary vascular resistance, WHO: world health organization

	Male PAH patients	Female PAH patients	Male controls	Female controls	*P* value
*N*	18	20	11	10	
Age, years	48.83 (34–61.42)	49.00 (34–54.5)	56 (38.17–59.54)	49.38 (29.44–65.25	0.8052
WHO group 1 (n (%))	18 (100)	20 (100)			
CO	5.200 (3.25–7.25)	3.740 (3.15–4.15)			0.0712
PVR, Wood units	9.040 (5.68–12.84)	11.42 (9.47–18.07)			0.1006
mPAP, mmHg	58.50 (33.75–72.5)	54.50 (48.50–59.75)			0.5372
6MWD, meters	326.2 (256.6–421.3)	273.4 (135–318.8)			0.1183
Therapy					0.9999
Monotherapy (n, (%))	0	0			
Double therapy (n, (%))	3 (21.4)	2 (18.1)			
Triple therapy (n, (%))	5 (35.7)	5 (45.5)			

### PAH patient plasma samples

PAH patients were recruited from the UCLA Pulmonary Vascular Disease Program. Blood was drawn at the time of right heart catheterization or clinic follow up visit. All individuals gave informed consent with approval from the institutional review board of the Office of the Human Research Protection Program (OHRPP) at UCLA. Blood was collected in EDTA tubes and centrifuged at 1200 g for 10 min at room temperature. Plasma was collected and stored in a -80 °C freezer prior to performing ELISA. Patient characteristics are described in Tables 2 and 3.

**Table 2:** UCLA plasma cohort characteristics stratified by sex. Available data is presented as median + interquartile range for continuous data, or as count + percentage for categorical data. Continuous data tested with Mann-Whitney test. Categorical data tested with fisher’s exact test. 6MWD: 6-minute walk distance. BNP: brain natriuretic peptide, DLCO: diffusing capacity of the lungs for carbon monoxide, mPAP: mean pulmonary arterial pressure, PCWP: pulmonary capillary wedge pressure, PVR: pulmonary vascular resistance, WHO: world health organization

	Male PAH patients	Female PAH patients	Male controls	Female controls	*P* value
*N*	22	25	7	6	
Age, years	61 (51–71)	51.50 (40.25–36.25)			0.002
WHO group (n (%))					0.002
1	5 (27.8)	20 (95.2)			
2	1 (5.6)	1 (4.8)			
3	3 (16.7)	0			
4	0	0			
DLCO, %	68.50 (31.68–83.13)	63 (36–79)			0.4487
PVR, Wood units	4.776 (3.171–6.383)	5.837 (3.105–13.35)			0.3424
mPAP, mmHg	36 (30–45)	41.5 (29.5–59.75)			0.2388
PCWP, mmHg	11 (8–14)	11 (8–13.95)			0.7977
RA, mmHg	8 (4–9)	9 (4.25–10)			0.5287
6MWD, meters	390 (282–424.5)	375 (306–469.5)			0.9734
BNP, pg/ml	145.5 (52.25–503.5)	43 (34.5–617)			0.2719
Therapy					0.2561
Monotherapy (n, (%))	3 (13.6)	6 (24)			
Double therapy (n, (%))	3 (13.6)	6 (24)			
Triple therapy (n, (%))	3 (13.6)	5 (20)			

**Table 3:** UCLA plasma cohort characteristics used for correlation with clinical characteristics. Available data is presented as median + interquartile range for continuous data, or as count + percentage for categorical data. Continuous data tested with Mann-Whitney test. Categorical data tested with fisher’s exact test. 6MWD: 6-minute walk distance. BNP: brain natriuretic peptide, DLCO: diffusing capacity of the lungs for carbon monoxide, mPAP: mean pulmonary arterial pressure, PCWP: pulmonary capillary wedge pressure, PVR: pulmonary vascular resistance, WHO: world health organization

	Male PAH patients	Female PAH patients	*P* value
*N*	12	24	
Age, years	68 (55–70)	54 (41.5–69.5)	0.1610
WHO group (n (%))			0.0804
1	7 (63.6)	20 (83.3)	
2	2 (18.2)	1 (4.2)	
3	2 (18.2)	2 (8.3)	
4	1 (14.3)	0	
DLCO, %	50.50 (82.98–31.55)	61.00 (32.5–79.45)	0.9074
PVR, Wood units	3.915 (3.41–4.73)	5.39 (2.82–10.29)	0.3145
mPAP, mmHg	30 (22–41)	38.00 (28.25–51)	0.09
PCWP, mmHg	11.00 (8–14)	11 (8–12.65)	0.9167
RA, mmHg	4 (2–8)	8 (4–10)	0.08
6MWD, meters	390.0 (217.5–512.5)	378.0 (312–471)	0.7961
BNP, pg/ml	80.50 (42.5–317.3)	43.00 (34–774)	0.6960
Therapy			0.268
Monotherapy (n, (%))	1 (8.3)	3 (12.5)	
Double therapy (n, (%))	3 (25)	7 (29.1)	
Triple therapy (n, (%))	1 (8.3)	7 (29.1)	

### ELISA

HGF was measured in human plasma using ELISA (R&D Systems #DHG00B) according to the manufacturer’s instructions. Absorbance was measured using a microplate reader (Synergy H1, BioTek).

### Cell culture

Primary pulmonary smooth muscle cells (PASMC) and pulmonary artery adventitial fibroblasts (PAFB) from both male and female PAH patients were obtained from PHBI and were grown in Vascular Smooth Muscle Cell Medium (Thermo #M231500) with smooth muscle growth supplement (SMGS, Thermo #S00725) and antibiotic/antimycotic (Thermo # 15240062). Patient characteristics are described in Table 4.

**Table 4:** Characteristics of PAH patient cells. 6MWD: 6-minute walk distance, CO: cardiac output, mPAP: mean pulmonary arterial pressure, PVR: pulmonary vascular resistance

	Male PASMC	Female PASMC	Male PAFB	Female PAFB
Age	40	24	57	29
PAH classification	IPAH	IPAH	IPAH	IPAH
WHO group	1	1	1	1
mPAP, mmHg	73	41	75	41
CO, L/min	3.1	4.44	7.47	5.4
PVR, Wood units	16.77	12.2	7.1	5
6MWD, meters	420	345	234.7	339
Therapy	Triple	Triple	Double	Triple

### Cell proliferation assay

PASMCs and PAFB were seeded in a 96-well plate (5000 cells/well) and treated accordingly with recombinant HGF protein (MyBiosource, #MBS2009596, 100ng/ml) or saline vehicle. CCK8 assay (Dojindo Laboratories, CK04) was performed 24 h after stimulation according to the manufacturer’s protocol.

### Immunofluorescence

Cells were grown on coverslips, fixed in 4% paraformaldehyde (Fisher), permeabilized with 10% Triton and blocked with 5% normal goat serum. Ki67 antibody (1:500; Millipore #AB9260), was incubated overnight at 4 °C and secondary antibody (Invitrogen #A21245) for 1 h at room temperature. Sections were mounted with Fluoromount G with DAPI (Thermo #00-4959-52) and imaged using a Nikon A1 confocal microscope. Cells positive for Ki67 as a proportion of total cell number were quantified in 5 random fields per coverslip per experimental replicate using ImageJ software (U.S. National Institutes of Health).

### Protein extraction, SDS-PAGE, and Western blotting

Proteins were isolated using RIPA lysis buffer (50 mM NaCl, 50 mM Tris pH 8, 1% NP- 40, 0.5% sodium deoxycholate, and 0.1% SDS, Sigma) containing protease and phosphatase inhibitors (Roche #04-906-845-001 and #118-3615-3001). Proteins were diluted in 4x Laemmli sample buffer (BioRad #161–0747), boiled, separated by SDS-PAGE, and transferred onto nitrocellulose membranes (BioRad #170–4270, TransBlot Turbo System, BioRad). Membranes were blocked with 5% bovine serum albumin (Sigma #A9647) and incubated with antibodies directed against cMET (R&D Systems #AF276) and GAPDH (Cell Signaling #2118). LI-COR IRDye-conjugated secondary antibodies were used for detection on the LI-COR Odyssey Infrared Imaging System. Bands were quantified using Image Studio Lite Version 5.2.

### Animals and *in vivo* PH model

The institutional Animal Research Committee approved all animal procedures (ARC-2010-045) and are according to current NIH guidelines. Male and female Sprague-Dawley rats (~ 250 gram) were purchased from Charles River. Rats were injected subcutaneously with Monocrotaline (MCT, 60 mg/kg, Sigma #C2401) or saline as control. A subset of rats was euthanized at day 7, 14, and 28 post-MCT injection.

To knockdown HGF in the lungs, starting at the day of MCT injection, rats received intratracheal instillations of siRNA targeting *Hgf* (2.5nM, Horizon, # A-089896-13-0050) or a scrambled control siRNA (Horizon, D-001910-01-50), every 3–4 days for a total of 5 instillations. Rats were euthanized at either 14 days or 28 days post-MCT injection.

At the end of each experiment, right ventricular systolic pressure (RVSP) was measured via open-chested catheterization (Millar Mikro-tip SPR-1000) under isoflurane anesthesia (2–3%). While under deep anesthesia, rats were euthanized via terminal blood draw and excision of heart and lungs. Right ventricle hypertrophy was assessed via Fulton Index (right ventricle weight/(left ventricle + intraventricular septum weight)). Echocardiography (Vevo 3100, VisualSonics) was performed to assess PH severity at baseline, day 14 and day 28. Pulsed-wave doppler imaging was acquired at the RV outflow tract in parasternal long-axis view from which pulmonary acceleration time (PAAT) was measured. RV fractional area change (RVFAC) was measured from the parasternal short-axis view at mid- papillary level.

### RNA isolation and RT-qPCR

Total RNA was isolated using Trizol (Thermo Fisher) according to the manufacturer’s instructions. Briefly, RNA was extracted from tissue using chloroform (ThermoFisher #364320025), isopropanol (MilliporeSigma #I9516), and 70% ethanol (MilliporeSigma #459844). Reverse transcription was performed using the High Capacity cDNA Reverse Transcription kit (Thermo, #4368814) and qPCR using Power Up SYBR Green Master Mix (Thermo, # A25779) on a BioRad CFX Connect PCR detection system. Primers are listed in Table 5.

**Table 5:** Primers sequences used for RT-qPCR. Hgf: hepatocyte growth factor, met: MET proto-oncogene, receptor tyrosine kinase, Rplp0: ribosomal protein lateral stalk subunit P0

Gene	Forward primer	Reverse primer
Human hgf	AAAGGACGCAGCTACAAGGG	CATGGAACTCCAGGGCTGAC
Human met	CCCACCCTTTGTTCAGTGTG	AGTCAAGGTGCAGCTCTCAT
Human rplp0	CAGGTGTTCGACAATGGCAG	ACAAGGCCAGGACTCGTTTG
Rat hgf	ACACAACAAACTTAGCTCATCGC	AGATGCCGGGCTGAAAGAAT
Rat rplp0	CTCAGTGCCTCACTCCATCA	CTTCCTTTGCTTCGACCTTG

### Masson trichrome staining

Rat lungs were fixed in 4% paraformaldehyde, embedded in OCT compound (Sakura #4583), and cryosections were cut to 7 μm. Tissue sections were stained with the Masson Trichrome Kit (Sigma #HT15) according to the manufacturer’s instructions. Images were made using an Axio Vert.A1 microscope (Zeiss). To assess medial wall thickness, at least 5 distal pulmonary arteries (< 100 μm) per animal were measured using ImageJ software (U.S. National Institutes of Health). External and internal diameter of transversally cut vessels as a ratio of the lumen in two perpendicular directions was calculated as described previously [21, 22]. Perivascular fibrosis was assessed as the ratio of total vessel wall as described previously [22]. 

### Statistical analysis

Distribution of the data was tested using the Kolmogorov–Smirnov normality test. Grubb’s test was used to test for significant outliers. Normally distributed data are presented as mean ± SEM and tested with Student’s t-test for 2 groups or one-way ANOVA with Holm-Bonferroni post-hoc correction for > 2 groups. Data with non-normal distribution are presented as median ± IQR and tested with Man-Whitney test for 2 groups or Kruskall-Wallis test with Dunn’s post-hoc correction for > 2 groups. Linear correlation was tested using Spearman’s r. Survival was tested with the Kaplan-Meier estimate. All analyses were performed using GraphPad Prism 10 software (San Diego, CA, USA).

## Results

### Discovery of HGF as a novel gene of which expression in the lungs is only upregulated in male PAH patients

**Fig. 1:**
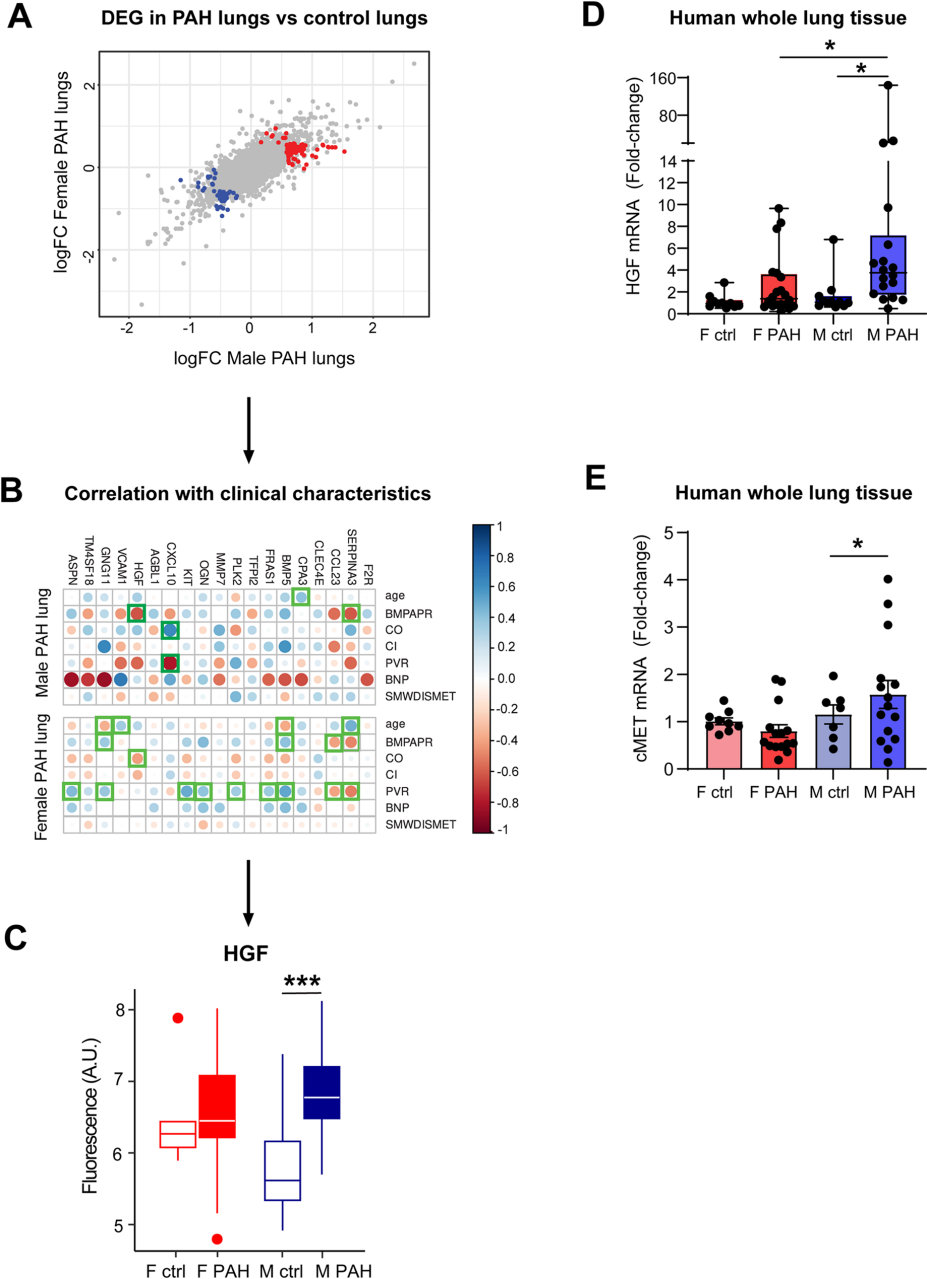
Bioinformatic discovery of HGF as a gene with elevated expression in lungs of male PAH patients. **(A)** The magnitude of expression changes in PAH vs. control lungs correlated between male and female PAH patients. Grey genes follow a similar linear correlation between sexes. Red genes are significantly upregulated in one sex compared to the other. Blue genes are significantly downregulated in one sex compared to the other. **(B)** Correlation of significant DEG with clinical characteristics in male and female PAH patients. Green squares represent statistically significant correlation. **(C)** Statistical analyses reveal Two-way ANOVA analysis was employed to identify genes that were: (1) not significantly differentially expressed in an overall analysis of control vs. PAH lungs, and (2) significantly differentially expressed between control and PAH only in one of the sexes. HGF was found as a DEG only in lungs of male PAH patients vs. male controls, but not females, nor in a sex-unbiased analysis of control lungs vs. PAH lungs. **(D)** Validation of HGF mRNA expression in human lungs by qPCR. Data presented as median ± IQR, *n* = 11–20 subjects/group, **p < 0.01, Kruskal-Wallis test with Dunn’s post-hoc correction. **(E)** cMET mRNA expression in human lungs by qPCR. Data presented as mean ± SEM, *n* = 7–15 subjects/group, *p < 0.05, ANOVA with Holm-Bonferonni post-hoc correction

To gain further insights into novel sex-specific factors in PAH, we leveraged a publicly available microarray dataset of the PHBI (GSE117261), comprising human lung tissues from 58 PAH patients and 25 healthy controls [19]. We correlated the fold-change magnitude of differentially expressed genes (DEG) between control and PAH lungs in males versus females (Fig. 1A). While most DEG exhibited a similar expression (grey points) in both males and females as shown by a linear correlation, a subset of DEG were significantly downregulated (blue points) or upregulated (red points) in one sex compared to the other (Fig. 1A). Among these sex-specific significant DEG, 19 correlated with clinical indicators of disease severity to an overall larger extent in male PAH patients than in female PAH patients (Fig. 1B). Of these 19 correlating DEG, 12 significantly correlated with at least one clinical disease severity parameter (Fig. 1B). This association suggests a potential link between the 12 DEG and sex differences in PAH pathophysiology. To prioritize genes for further investigation, we next applied a two-way ANOVA, focusing on genes that were: (1) not differentially expressed between control versus PAH lungs when both sexes were combined, but (2) significantly differentially expressed between control and PAH in only one sex when analyzed separately. This stringent approach identified *HGF* (hepatocyte growth factor) and *GNG11* (Guanine nucleotide-binding protein subunit gamma-11) as top two candidate genes (Fig. 1C & Supp. Figure 1). Both *HGF* and *GNG11* were significantly upregulated in PAH compared to control lungs in male subjects but were not differentially expressed between control and PAH lungs in female subjects (Fig. 1C & Supp. Figure 1). To experimentally validate our bioinformatic analyses, we examined expression of both HGF and GNG11 in lung tissue from male and female PAH patients, as well as control subjects. GNG11 mRNA expression was undetectable by qPCR. However, we confirmed significant HGF mRNA upregulation in the lungs of male PAH patients, but not female PAH patients compared to their corresponding controls, consistent with our bioinformatics discovery (Fig. 1D). Therefore, we focused on the role of HGF *as a* gene with sex-specific expression in lungs of PAH patients.

HGF binds to the cMET receptor [23]. Thus, we next assessed whether there is also a sex-specific difference in cMET expression in PAH lungs and found that indeed cMET mRNA is exclusively upregulated in male PAH patients, but not female PAH patients (Fig. 1E).

### Elevated plasma HGF correlates with favorable clinical characteristics only in male PAH patients

**Fig. 2:**
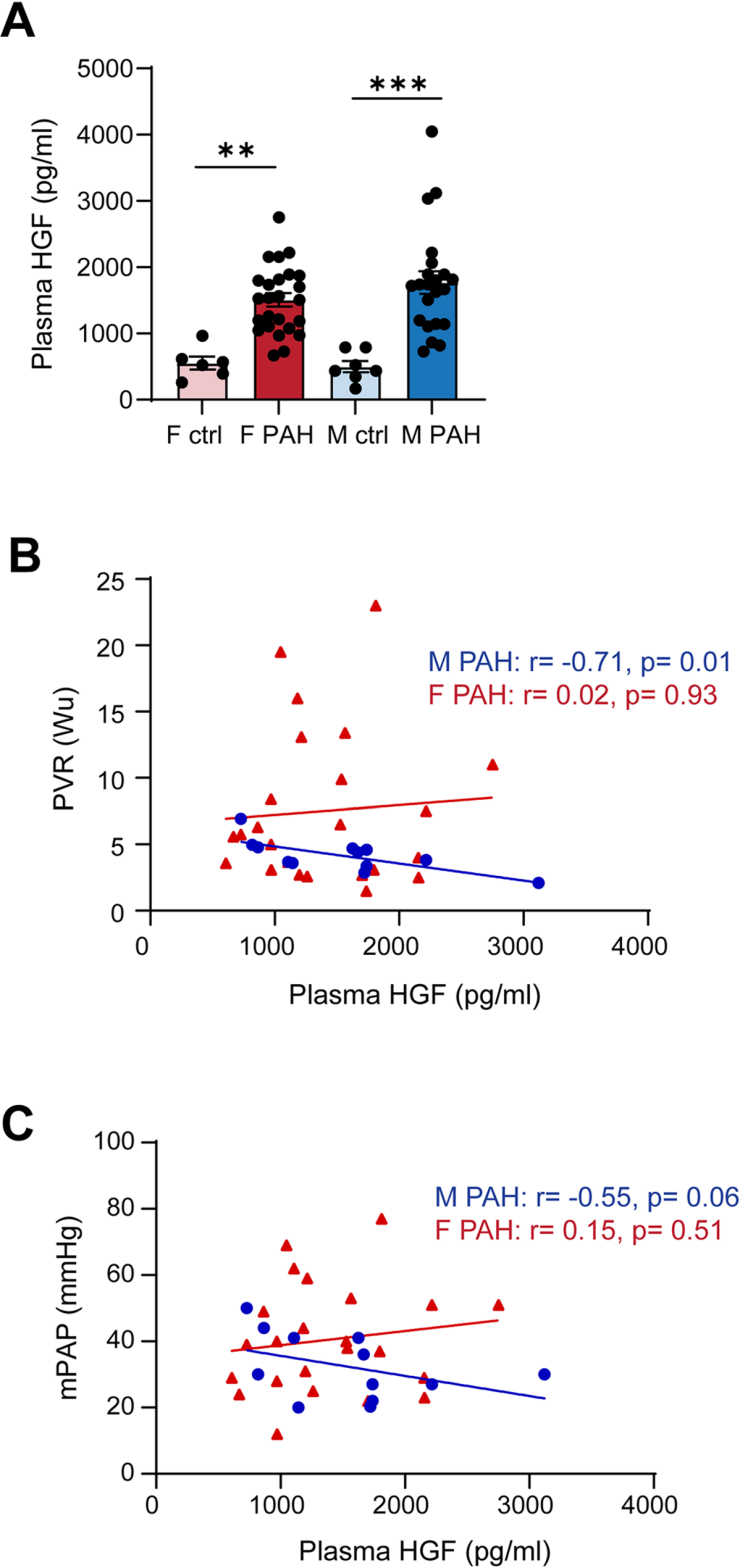
Elevated plasma HGF correlates with favorable clinical characteristics only in male PAH patients. **(A)** HGF in plasma samples from control subjects and PAH patients measured by ELISA. *N* = 6–7 controls/group, *n* = 22–25 PAH patients/group. Data presented as mean ± SEM, **p < 0.01, ***p < 0.001, ANOVA with Holm-Bonferroni post-hoc correction. **(B)** Correlation between plasma HGF and pulmonary vascular resistance in PAH patients. **(C)** Correlation between plasma HGF and mean pulmonary arterial pressure in PAH patients. Each point represents one patient. Red: female PAH patients, *n* = 24 Blue: male PAH patients, *n* = 12. Spearman’s r

HGF may be secreted into the circulation to exert autocrine and paracrine effects. Additionally circulating HGF has been shown to serve as a biomarker for disease severity in cardiovascular disease. As such, we next measured plasma HGF in an independent cohort of PAH patients but did not observe sex differences in plasma HGF levels as plasma HGF is upregulated both in male and female PAH patients compared to their respective controls (Fig. 2A). Since our bioinformatic analysis revealed sex differences in the correlation between lung HGF expression and clinical characteristics in PAH patients, we examined whether there is a correlation between plasma HGF levels and pulmonary vascular resistance (PVR) or mean pulmonary arterial pressure (mPAP) in male and female PAH patients. In male PAH patients, there was a significant negative correlation between plasma HGF with PVR (Fig. 2B) and a trend toward negative correlation with mPAP (Fig. 2C). In contrast, there was no significant correlation between plasma HGF levels and PVR or mPAP in female PAH patients (Fig. Figure 2A, B). Taken together, these data indicate that HGF in the lungs may be upregulated as a compensatory mechanism to counteract PH disease progression and that plasma HGF may serve as a sex-specific biomarker for clinical severity in PAH patients.

### HGF is expressed in vascular SMC and FB in the lung and recombinant HGF suppresses proliferation of SMC and FB to a significantly greater extent in male than in female cells

**Fig. 3:**
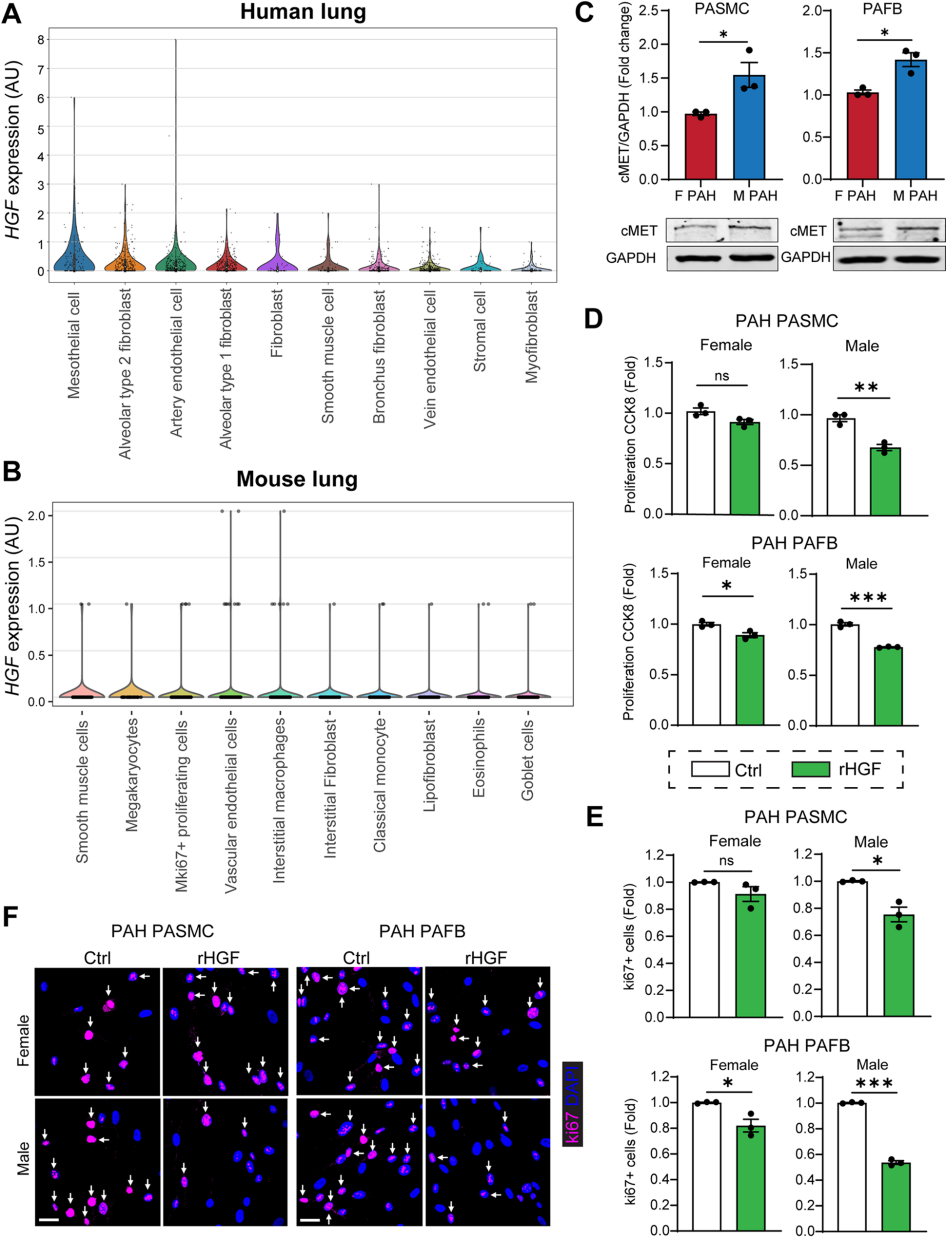
HGF is expressed in vascular mesenchymal cells in the lung and exerts larger effects in male cells than female cells. **(A)** Violin plots showing the top 10 cell types in human lungs highest in HGF RNA expression. Each point represents one donor. From the Human Lung Cell Atlas. **(B)** Violin plots showing the top 10 cell types in mouse lungs highest in HGF RNA expression. Each point represents one cell. From Tabula Muris Senis Atlas. **(C)** Western blot assessment of HGF receptor cMET expression in human primary PASMC and PAFB isolated from female and male PAH patients. **(D)** Proliferation in PASMC and PAFB stimulated with recombinant HGF protein by CCK8 assay and by ki67 immunofluorescence **(E**,** F**, scale bar is 10 micron**)**. Data presented as mean ± SEM, *n* = 3 independent experiments/group, *p < 0.05, **p < 0.01, ***p < 0.001, Student’s t-test

We next assessed which cell types in the lung express HGF by analyzing publicly available single cell atlases [24, 25]. Both in human lungs (Fig. 3A) and mouse lungs (Fig. 3B), the top 10 cell types with highest HGF RNA expression were enriched for mesenchymal cells. Specifically, smooth muscle cells (SMC) and fibroblasts (FB) were among the top-enriched cell types for HGF expression (Fig. 3A, B).

Since our data indicate that higher HGF expression in the lungs is associated with more favorable clinical characteristics in male PAH patients, and since PASMC and PAFB dysfunction is a hallmark of PAH development, we next assessed the effect of HGF on PASMC and PAFB isolated from male and female PAH patients. We first assessed cMET receptor expression by Western blot and found that both male PASMC and PAFB express significantly higher levels of cMET than female cells (Fig. 3C). Next, we assessed the effect of recombinant HGF on proliferation of PASMC and PAFB via two different methods: dehydrogenase activity assay (Fig. 3D) and expression of proliferation marker ki67 (Fig. 3E, F). Both methods showed that recombinant HGF inhibits proliferation in PASMC and PAFB isolated from PAH patients of both sexes but had a significantly larger inhibitory effect in cells isolated from male than female. Thus, our data indicate a sex difference in the protective role of HGF against PASMC and PASMC dysfunction in PAH.

### Lung HGF transcripts are upregulated to a higher extent and for a longer duration in male compared with female rats in response to PH

**Fig. 4:**
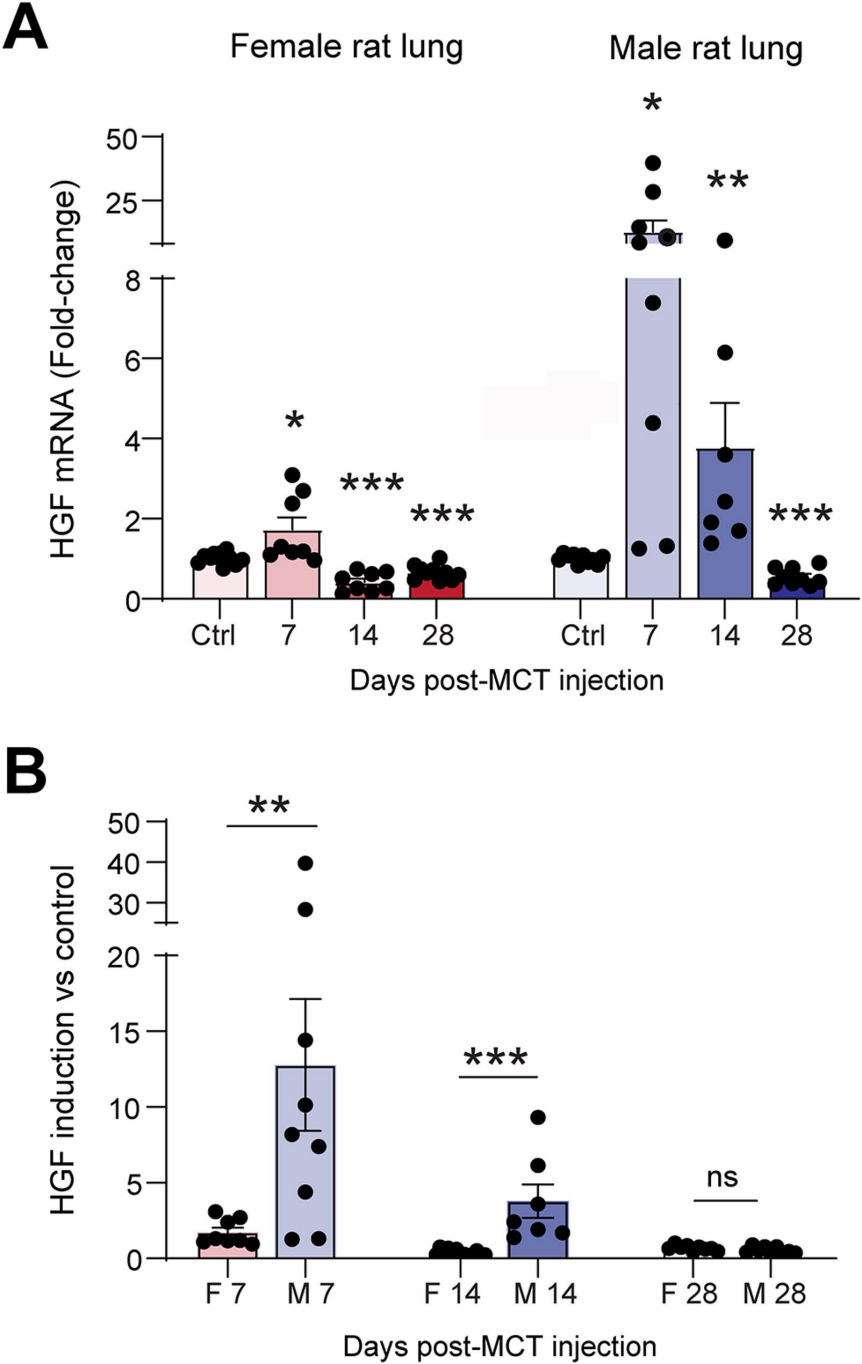
HGF is increased to a higher and longer extent in lungs of male than female rats in response to PH. HGF mRNA expression assessed by qPCR. Data presented as mean ± SEM, *n* = 8–11 animals/group. **(A)** *p < 0.05, **p < 0.01, ***p < 0.001 vs. respective control, ANOVA with Holm-Bonferroni post-hoc correction. **(B)** Comparison of HGF mRNA expression change between female vs. male rats per time point, **p < 0.01, ***p < 0.001, Student’s t-test

Since HGF is upregulated in male PAH lungs and higher HGF expression levels correspond with favorable clinical characteristics and reduced proliferation of PASMC and PAFB, we hypothesized that HGF may act as a compensatory response against PH development in males. We employed the Monocrotaline (MCT) model of PH in male and female rats and measured lung HGF expression at different timepoints of PH development (Fig. 4A). Strikingly, HGF lung expression was significantly upregulated already at 7 days after MCT injection in both male and female rats, but the upregulation was to a significantly greater extent in males (~ 13-fold) than in females (~ 2-fold; Fig. 4B). Additionally, HGF expression remained significantly elevated (~ 4 fold) at day 14 days post-MCT only in males, but not in females. Interestingly, in both sexes in the MCT and the Sugen/Hypoxia models of PH, HGF expression in the lungs was downregulated at the disease endpoints at 4 and 5 weeks respectively (Supplemental Fig. 2).

Together, these data indicate sex-specific differences in the early compensatory upregulation of HGF in the lungs in response to PH stimuli.

### Blocking early HGF upregulation promotes PH development in male rats

**Fig. 5:**
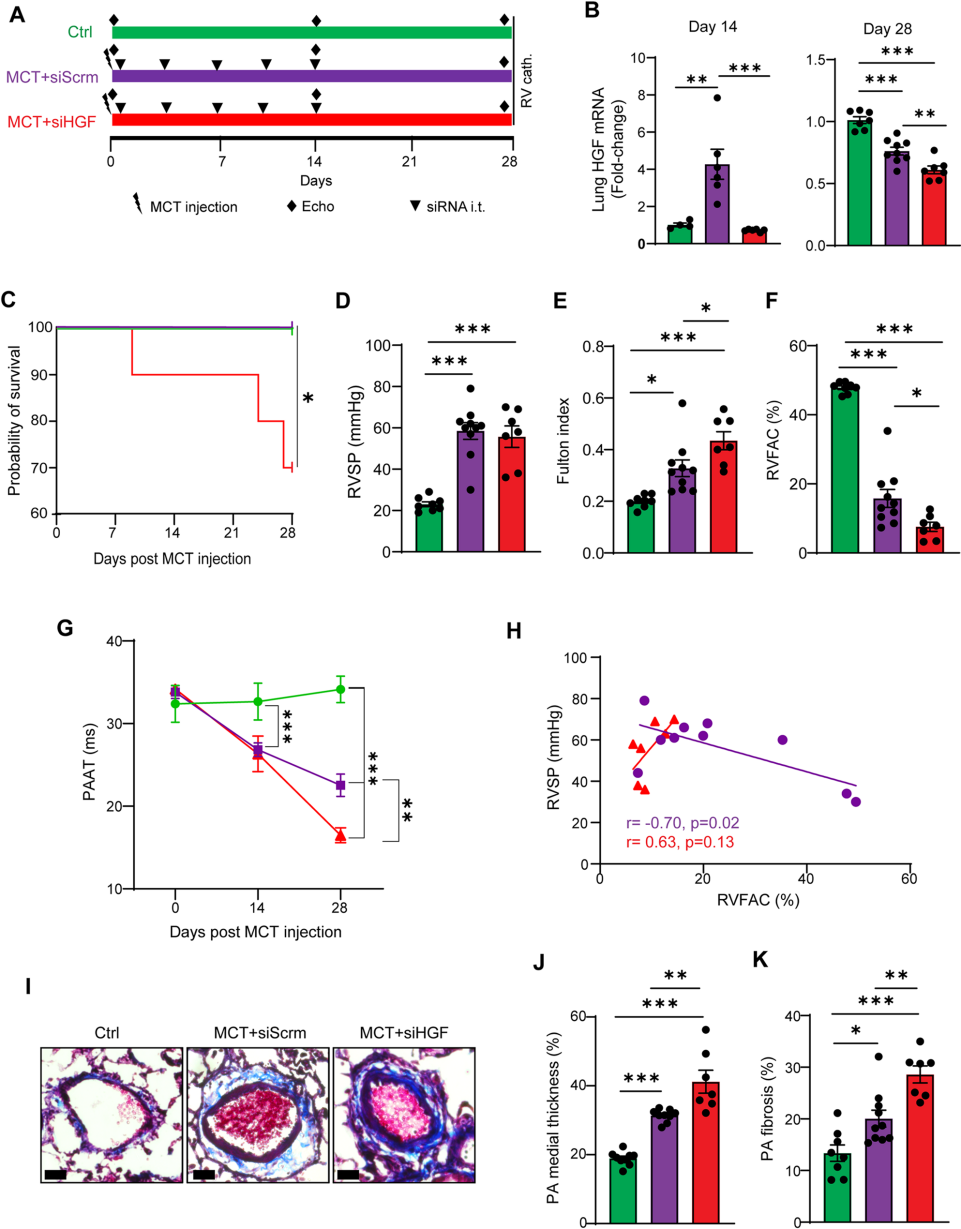
Blocking early HGF upregulation promotes PH development in male rats. **(A)** Experimental timeline. Green bars: control rats, purple bars: MCT rats treated with scrambled siRNA, red bars: MCT rats treated with HGF siRNA **(B)** Knockdown efficiency of HGF in the lungs at 14 and 28 days after MCT injection. **(C)** Kaplan-Meier showing survival after MCT injection. **(D)** Right ventricular systolic pressure on day 28 post-MCT as measured by catheterization. **(E)** RV hypertrophy index at 28 days post-MCT. **(F)** RV function as determined by fractional area change by echocardiography at 28 days post-MCT. **(G)** Longitudinal follow-up of pulmonary artery acceleration time upon MCT injection as assessed by echocardiography. **(H)** Correlation between right ventricular pressure and right ventricular fractional area change per animal. **(I-K)** Medial thickening and perivascular fibrosis of distal pulmonary arteries in rats, as assessed by Masson trichrome staining. Scale bars 20 μm. **(B**,** D-G**,** J**,** K)** Data presented as mean ± SEM, *n* = 7–10 animals/group, *p < 0.05, **p < 0.01, ***p < 0.001, ANOVA with Holm-Bonferroni post-hoc correction. **(H)** Data presented as mean, Spearman’s r

To assess the causal role of HGF in PH development in male rats, we next assessed whether the early upregulation of HGF expression in the lungs may protect against PH development. As such, we administered HGF siRNA intratracheally in rats immediately upon MCT insult (day 1 to 14 post MCT, Fig. 5A) and observed significantly lower HGF mRNA expression in the lungs of siHGF rats compared to siScrm rats at both 14 and 28 days post-MCT injection (Fig. 5B). We found that siHGF-treated MCT had significantly lower survival rate than siScrm-treated MCT rats (Fig. 5C). Interestingly, there was no significant difference in RVSP between siScrm and siHGF rats (Fig. 5D), while siHGF rats did exhibit significantly higher RV hypertrophy (Fig. 5E), worsened RV function based on significantly lower RVFAC (Fig. 5F), and worsened pulmonary vascular function as observed by significantly lower PAAT (Fig. 5G) throughout the experiment. To assess whether the lack of significant differences in RVSP between siScrm and siHGF MCT rats might reflect decompensated RV failure, where the RV cannot sustain elevated pressure due to poor contractility, we correlated RVSP with RVFAC in the MCT-treated animals. Strikingly, we found opposite correlations between siScrm and siHGF rats (Fig. 5G) where RVSP and RVFAC negatively correlated in siScrm rats, but positively correlated in siHGF rats, suggesting that siHGF rats may be undergoing RV failure. Consistent with this, histological analyses revealed that siHGF rats exhibited more severe pathological PA vascular remodeling with enhanced medial thickening (Fig. 5I, J) and enhanced perivascular fibrosis as compared to siScrm-treated MCT rats (Fig. 5I, K). These data are in line with the pro-proliferative effects of HGF silencing in human PASMC and PAFB (Supplemental Fig. 3).

Together, these data indicate a causal role for HGF in PH, indicating that early HGF upregulation in males protects against PH development.

## Discussion

The incidence of PAH is much higher in female patients, up to a 4:1 ratio [5], indicating that males may have a protective factor that could help elucidate the pathogenesis of PAH. In this study, we present a novel gene, HGF, that might at least partly contribute to the lower susceptibility to PAH in males. Our data shows that HGF transcript expression is only upregulated in lungs of male PAH patients compared to male controls but does not change in female PAH patients vs. female controls. Strikingly, we found that elevated plasma HGF correlates with favorable clinical characteristics only in male PAH patients. We also show that HGF is mainly expressed in vascular SMC and FB in the lung and found that recombinant HGF inhibits proliferation of PASMC and PAFB to a greater extent in cells isolated from male PAH patients compared to female PAH patients. In the monoctrotaline rat PH model, lung HGF is upregulated to a higher and longer extent in male compared to female rats and importantly, we found that early HGF upregulation in the lungs is essential for protection against PH development in male rats.

Here, we show for the first time a sex difference in lung HGF expression in PAH patients. Elevated expression of HGF in pulmonary arterioles and in RV from PAH patients versus control subjects has been previously reported, however sex differences were not assessed [26, 27]. Sex differences in HGF expression have previously been shown in serum of hepatocellular carcinoma patients [12], synovial fluid from osteoarthritis patients [13], and serum of ankylosing spondylitis patients [14], with male patients exhibiting elevated HGF levels. We furthermore, show that elevated plasma HGF correlates with favorable clinical characteristics in male PAH patients, shows no significant correlation in female PAH patients, indicating the potential of HGF as a sex-specific biomarker in PAH patients. In accordance with our findings, serum HGF levels significantly associated with disease progression in male ankylosing spondylitis patients, but not female [14]. On the other hand, HGF expression in pleural effusion had a higher favorable diagnostic efficacy for tuberculosis in young female patients, as compared to young male patients [28]. 

Understanding the sex-dependent regulation of HGF expression is crucial for a complete understanding of its biological functions. Regarding sex hormones, estradiol was shown to promote HGF expression in various tissues [29–31]. In mice, the HGF promoter has a functional estrogen receptor element (ERE) and ERs can enhance HGF promoter activity via direct ERE binding as well as trans-repression of transcription factor COUP-TF. Although the HGF promoter lacks androgen receptor response elements (ARE), androgens have been reported to indirectly repress HGF and its signaling pathway. Regarding sex chromosomes, a recent study employing single cell RNAseq in lymphoblastoid cells found that most genes expressed in a sex-biased manner are targets of transcription factors that are differentially expressed between sexes [32]. Among these, FOSL1, of which expression is dependent on numbers of X chromosomes, was identified as one of the transcription factors making the largest contribution to this sex bias [32]. FOSL1 was demonstrated to be expressed higher in male cells [32]. FOSL1 is a component of the AP-1 transcription factor complex which has been reported to directly upregulate HGF expression. Thus, we speculate that enhanced levels of AP1 subunit FOSL1 in males could underlie higher HGF expression in males. However, future research is warranted into the mechanisms underlying the sex-dimorphic expression of HGF in PAH.

In line with our data, a large proteomic profiling study of PAH patient plasma by Amsallem et al. showed that there was no difference between plasma HGF levels in male PAH patients compared to female PAH patients [27]. While our plasma HGF protein and lung HGF transcript data contrast in showing a sex difference in HGF patients, it’s worthwhile noting that HGF is a secreted protein, and we hypothesize that its plasma concentration reflects the sum of secretion rates from various tissues and clearance in the circulation. For instance, HGF is upregulated in the liver and kidneys [33, 34] upon disease and both kidney and liver dysfunction are remarkably prevalent in PH patients [35, 36], implying that the liver and kidneys may contribute to elevated plasma HGF in PAH patients. Additionally, HGF expression in the RV is shown to be upregulated in PAH by several studies which have alluded to the RV as one of the important sources of plasma HGF. Interestingly, our data does show that plasma HGF negatively correlates with clinical markers of PAH severity - PVR and mPAP - in male PAH patients but shows a positive correlation trend in female PAH patients. These findings indicate that while plasma HGF levels are similar between PAH patients of both sexes, its clinical relevance appears to be sex dependent. Our data underscore that a lack of difference in absolute plasma HGF concentration does not preclude sex-specific importance, further reinforcing the value of sex-stratified approaches in PAH biomarker discovery. Interestingly, Amsallem et al. reported that higher HGF plasma levels were associated with worse PAH severity with a maladaptive RV phenotype, and were predictive of 3-year clinical worsening [27]. This discrepancy with the proteomic study by Amsallem et al., may be explained by the fact that there is a significantly higher proportion of female PAH patients than male PAH patients analyzed, which may drive the positive correlation between plasma HGF and PAH severity parameters. Indeed, in our study, plasma HGF had no significant correlation with PVR or mPAP when male and female PAH patients were combined in the analysis. While a limitation of our study is the relatively small sample size of plasma samples with clinical characteristics, especially from male PAH patients, our data indicate the importance of sex-stratification in assessment of plasma HGF as a clinical biomarker in PAH.

We show for the first time the causal relationship between HGF and PH disease development by inhibiting the early MCT-induced HGF upregulation in the lungs via siRNA in the first two weeks after MCT injection, before the onset of MCT-induced PH symptoms. Our data show that blunted early HGF upregulation worsens PH severity in male rats, indicating that HGF is indeed upregulated as a protective mechanism against PH development. A limitation of our current study is that the functional HGF silencing experiments were conducted exclusively in male rats since male rats exhibited the most pronounced induction of HGF by MCT. While these results establish a causal link between HGF and MCT-induced PH in males, future studies are warranted to determine if a similar causal relationship exists in female rats as well. We however speculate that silencing HGF in female rats will also promote PH development since we show that HGF silencing in human PASMC and PAFB promoted proliferation in cells from both male and female. Our data indicate that directly depleting the endogenous HGF pool appears to critically regulate proliferation in both sexes.

In line with our data, the therapeutic potential of HGF has previously been shown. Several studies have reported that HGF can rescue MCT-induced PH in male rats by improving RVSP, pulmonary and RV hemodynamics and RV hypertrophy [15–18, 37]. We show that HGF can inhibit PASMC and PAFB proliferation and in line with our data, intratracheal delivery of HGF lentiviral particles was shown to inhibit PASMC proliferation and enhanced angiogenesis in the lungs, leading to increased numbers of effective perfusion vessels in MCT rats [16]. Additionally, viral overexpression of HGF via the PA was shown to attenuate MCT-induced PA wall remodeling with lower numbers of proliferating PASMCs and higher numbers of apoptotic PASMC, concomitant with decreased TGFb1 expression in the lung [15]. Our data demonstrate that HGF has a more pronounced inhibitory effect on proliferation in male PASMC and PAFB than female cells. Accordingly, we show that both whole lungs and PASMC and PAFB from male PAH patients express higher levels of HGF receptor cMET mRNA than female PAH tissue. While not much is known about sex-specific cMET expression, it was reported that cMET expression was higher in pituitary and colorectal tumors, from female patients compared to male [38, 39]. Future research is warranted to characterize sex-specific cMET expression and activity in PASMC and PAFB in PAH.

Our data shows that in both human and mouse lungs, HGF is most highly expressed in mesenchymal cells including SMC and FB. Highlighting the importance of HGF in pulmonary mesenchymal cells, it has previously been reported that injection of MCT rats with mesenchymal stem cells genetically modified to overexpress HGF improved RVSP, PA vascular remodeling, RV hypertrophy and fibrosis [18]. Additionally, HGF mesenchymal stem cells were shown to prevent MCT-induced disorganization of gap junctions in RV myocytes, thus leading to better RV function. However, our single cell atlas queries have also shown that HGF is expressed in non-mesenchymal cell types in the lungs, such as endothelial cells and immune cells. Since endothelial dysfunction [40] and altered immune cell dynamics [41, 42] also play a major role in PH development, future studies focusing on the relative importance of HGF expression in each respective pulmonary vascular cell type are imperative. For instance, it has been shown that systemic injection of recombinant HGF attenuates pro-inflammatory NF-κB signaling in lungs of MCT rats [17].

While less susceptible to developing PAH, male PAH patients have worse outcomes than female PAH patients, mainly reflected by worse RV function [43, 44]. This is known as the PAH sex paradox. In our study we have only measured HGF expression in the lungs of PAH patients and PH rats, and not in the RV. However, publicly available RNAseq dataset GSE198618 does not reveal significant differences in HGF expression in the RV of male vs. females, neither in controls nor PH patients. Adenoviral HGF overexpression has previously been shown to prevent RV remodeling in rats with MCT-induced PH [15, 45]. Thus, while upregulation of HGF in the lungs may partly explain why males are less susceptible to develop PH, the lack of HGF upregulation in the RV may partly explain why male have worse RV function and outcomes. Interestingly, female PAH patients exhibit no HGF upregulation in neither lungs nor RV, indicating that HGF may only contribute to the male-specific arm of the PAH sex paradox.

Taken together, our findings indicate that higher levels of HGF may at least partially contribute to the lower incidence of male PAH patients. As traction is being gained for leveraging large-scale databases and multiomics [46], and the importance of considering sex as a biological variable in preclinical pulmonary research is recognized [47], future studies hold high potential to further elucidate the mechanisms underlying the sex paradox in PAH.

## Conclusions

HGF expression in the lungs may be upregulated to counteract PAH disease progression by inhibiting proliferation of PASMC and PAFB. Since HGF expression is only upregulated in lungs of male PAH patients and higher plasma HGF correlates with favorable clinical characteristics only in male PAH patients, elevated HGF in males may at least partially contribute to the lower incidence of male PAH patients.

## Supplementary Information

Below is the link to the electronic supplementary material.

Supplementary Material 1

## Data Availability

This manuscript does not report data generation or analysis. All analyzed datasets are publicly available and GSE numbers and references are provided.
