# Supplementary material for: Hepatocyte growth factor may contribute to male protection against pulmonary arterial hypertension
Source: Biol Sex Differ. 2026 Mar 1;17:64. doi: 10.1186/s13293-026-00852-6 (PMC13059264; doi:10.1186/s13293-026-00852-6)
Supplement: Supplementary file 1 — Supplementary Material 1 [file 13293_2026_852_MOESM1_ESM.docx]

**Hepatocyte Growth Factor May Contribute To Male Protection Against Pulmonary Arterial** **Hypertension**

Lejla Medzikovic PhD^1#^, Gregoire Ruffenach PhD^1,2^*, Ateyeh Dehghanitafti MD^1^*, Brenda Wong BS^3^, Ashley Ryder^1^, Mohammadreza Hatamnejad MD MPH^1^, Wasila Sun BS^1^, Leana Esdin^1^, Joshua Eghbali^1^, Adam Brownstein MD PhD^3^, Asif Razee PhD^1^, Soban Umar MD PhD^1^ , Jason Hong MD PhD^3^, Mansoureh Eghbali PhD^1^

^1^ Division of Molecular Medicine, Department of Anesthesiology and Perioperative Medicine, David Geffen School of Medicine, University of California Los Angeles, USA

^2^ Paris Saclay University, Inserm, UMR-S 999, Pulmonary Hypertension: Physiopathology and Therapeutic Innovation, AP-HP, Hôpital Bicêtre, Hôpital Marie Lannelongue (Groupe Hospitalier Paris Saint Joseph), ERN-LUNG, Le Plessis Robinson, France

^3^ Division of Pulmonary and Critical Care Medicine, David Geffen School of Medicine, University of California Los Angeles, USA

*Equal contribution

#Corresponding author: Dr. Mansoureh Eghbali, Division of Molecular Medicine, Department of Anesthesiology and Perioperative Medicine, David Geffen School of Medicine, University of California Los Angeles, BH-550 CHS, Los Angeles, CA, 90095-7115, USA, [meghbali@ucla.edu](mailto:meghbali@ucla.edu)

**SUPPLEMENTAL DATA**


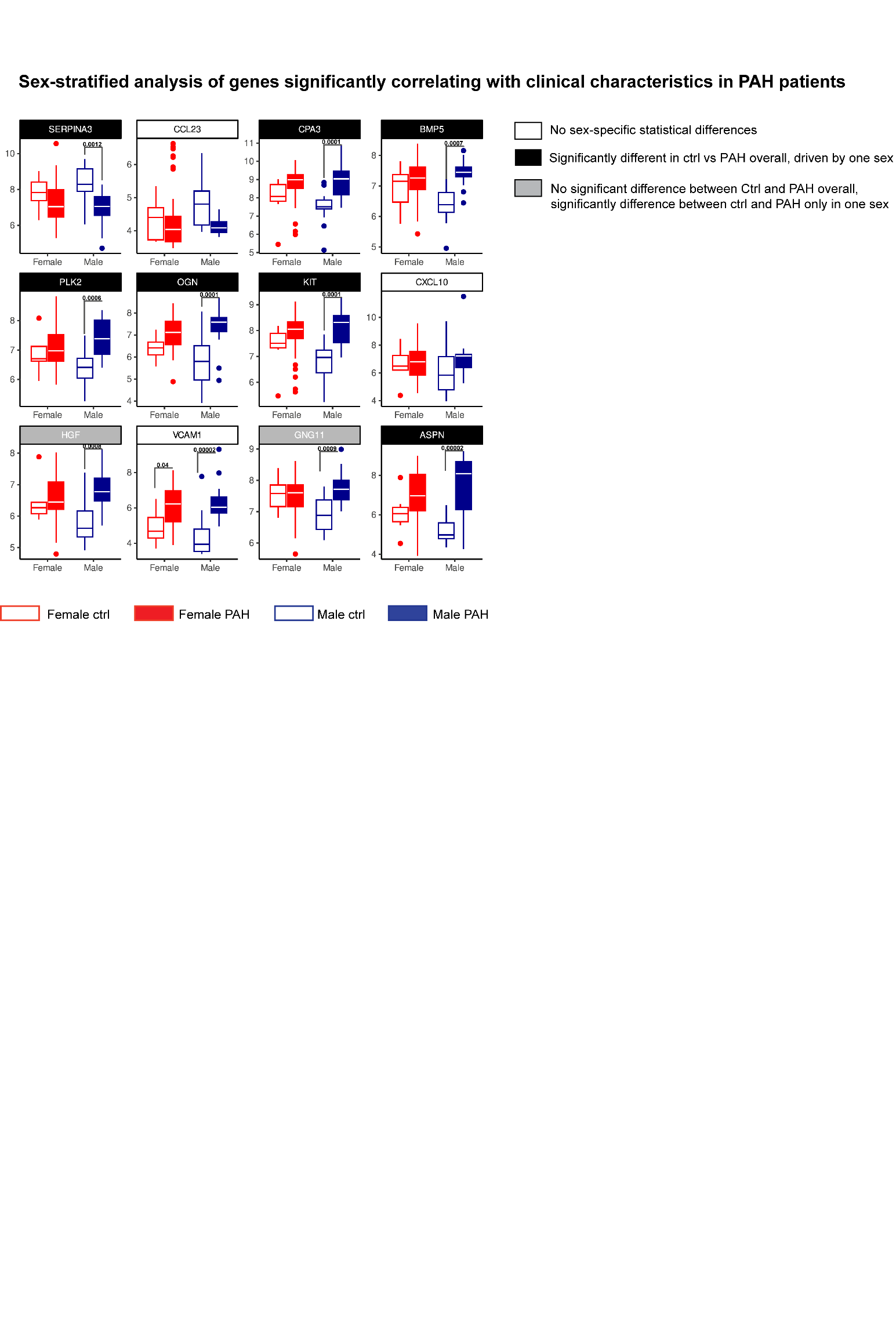


**Supplemental Figure 1. Sex stratified analysis of genes significantly correlating with clinical characteristics in PAH patients.** Analyzed from the publicly available microarray dataset GSE117261 from the Pulmonary Hypertension Breaktrough Initiative (PHBI) consisting of lungs from 58 PAH patients and 25 control lung tissues, together with all available clinical information. A two-way ANOVA analysis was employed to identify genes with expression differences in directions annotated with white, gray and black boxes.


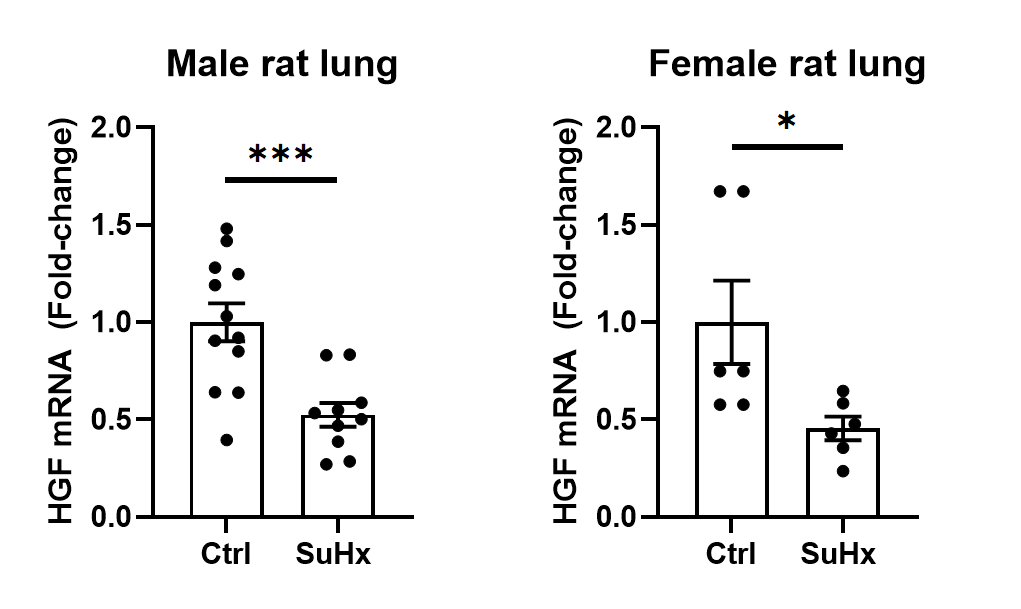


**Supplemental Figure 2. Downregulated HGF expression in the lungs of both female and male Sugen/Hypoxia PH rats.** PH was induced by s.c. injection of 20mg/kg Sugen followed by three weeks of hypoxia (10%O2) and two weeks of normoxia (21% O2). Data presented as mean±SEM, *p<0.05, **p<0.001, Student’s t-test.

**
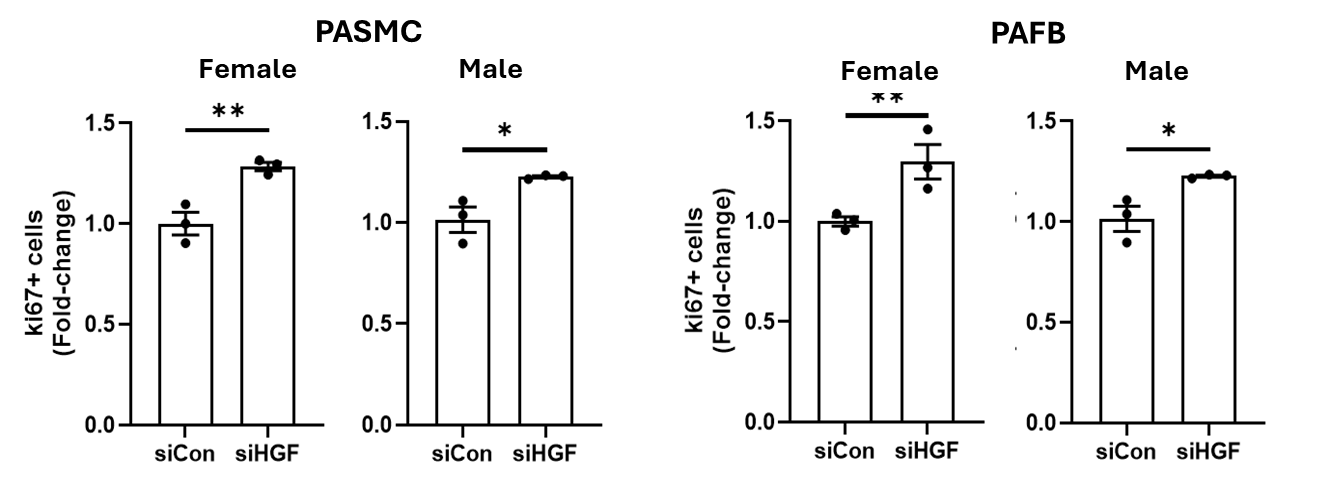
**

**Supplemental Figure 3. HGF silencing promotes proliferation in PASMC and PAFB from both sexes.** HGF was silenced via siRNA-mediated knockdown (40nM, Dharmacon, L-006650-00-0005 & D-001810-10-05) for 24h. Ki67+ cells were assessed by immunofluorescence. Data presented as mean±SEM,N=3 independent experiments, *p<0.05, **p<0.001, Student’s t-test.
